# Methods of policy monitoring in physical activity promotion: a systematic review across different levels of government: Sven Messing

**DOI:** 10.1093/eurpub/ckac129.269

**Published:** 2022-10-25

**Authors:** S Messing, A Tcymbal, K Abu-Omar, D Richardson, P Gelius

**Affiliations:** Department of Sport Science and Sport, Friedrich-Alexander-Universität Erlangen-Nürn, Erlangen, Germany

## Abstract

**Background:**

Even though the importance of policy monitoring in public health has increased in the last decades, there is still a lack of understanding what different approaches of policy monitoring exist and which methodology they employ. In order to address this research gap, this review attempts to provide a comprehensive overview about the methods of policy monitoring in the field of physical activity promotion.

**Methods:**

A systematic search was conducted in five scientific databases, using the terms “physical activity”, “policy” and “monitoring” and their variations. In total, 12.963 studies were identified and, after the elimination of duplicates, screened independently by two reviewers. During full text analysis, information on the methods applied for policy monitoring was extracted and studies were categorized based on their key characteristics (monitoring tool, policy level, and setting).

**Results:**

The search yielded in a total of 112 studies that were structured into seven categories: Report Cards on Physical Activity for Children and Youth, HEPA Monitoring Framework, HEPA Policy Audit Tool, national policies, subnational policies, school setting, and childcare setting. Across all categories, policy monitoring focused mainly on national level policies in a single country. Differences were identified with regards to the level of government involvement which allowed to differentiate between research-driven approaches (little or no government involvement), government-driven approaches (led by governments), and co-production approaches (strong collaboration between researchers and governments).

**Conclusions:**

Research-driven, government-driven and co-production approaches have different strengths and weaknesses with regards to the monitoring of policies. Awareness needs to be raised regarding the implications of these approaches, and more research is needed to analyse the impact of policy monitoring on policy-making in public health.

